# Pedi-IKDC or KOOS-child: which questionnaire should be used in children with knee disorders?

**DOI:** 10.1186/s12891-019-2600-6

**Published:** 2019-05-22

**Authors:** Charlotte A. van der Velden, M. C. van der Steen, Jens Leenders, Florens Q. M. P. van Douveren, Rob P. A. Janssen, Max Reijman

**Affiliations:** 1 Orthopaedic Center Máxima, Máxima Medical Center, Postbus 90052, 5600 PD Eindhoven, The Netherlands; 2 Department of Orthopaedic Surgery, Catharina Hospital Eindhoven, Postbus 1350, 5602 ZA Eindhoven, The Netherlands

**Keywords:** Translation, Validation, Patient reported outcome measures, Adolescents

## Abstract

**Background:**

The Pedi International Knee Documentation Committee (IKDC) and Knee Injury and Osteoarthritis Outcome Score (KOOS) child are validated questionnaires for children with knee disorders. The aim of this study was to translate these questionnaires in Dutch and to recommend which questionnaires should – based on their psychometric properties – be used in clinical practice.

**Methods:**

The English Pedi-IKDC and KOOS-Child were translated by the forward-backward procedure. Subsequently, content validity of the Pedi-IKDC and KOOS-Child was evaluated by both patients (*n* = 18) and experts (*n* = 18). To evaluate construct validity and interpretability participants with knee disorders (*n* = 100) completed the Numeric Rating Scale Pain, Lysholm Knee Scoring Scale, EuroQol-5 Dimension, Pedi-IKDC and KOOS-Child at baseline. Participants completed the anchor question, Pedi-IKDC and KOOS-child two weeks (*n* = 54) and one year (*n* = 71) after baseline, for evaluating the test-retest reliability and responsiveness. Psychometric properties were interpreted following the COnsensus-based Standards for the selection of health Measurement INstruments (COSMIN) criteria.

**Results:**

The Pedi-IKDC showed adequate test-retest reliability (intraclass correlation coefficient (ICC) 0.9; standard error of measurement (SEM) 8.6; smallest detectable change (SDC) 23.8), adequate content validity (> 75% relevant), adequate construct validity (75% confirmed hypotheses), low floor or ceiling effects (scores between 5 and 95) and adequate responsiveness (> 75% confirmed hypotheses). The KOOS-Child showed an adequate test-retest reliability (ICC 0.8–0.9; SEM 8.9–16.9; SDC 24.7–46.9), adequate content validity (> 75% relevant, except KOOS-Child subscale ADL), adequate construct validity (75% confirmed hypotheses), low floor and ceiling effects (scores between 5 and 95, except KOOS-Child subscale activities of daily living and Sport/play) and moderate responsiveness (40% confirmed hypotheses).

**Conclusions:**

The Pedi IKDC showed better psychometric properties than the KOOS-Child and should therefore be used in children with knee disorders.

## Background

The knee is commonly injured in children and adolescent. The most contributing factors of knee injuries in these age groups are sport activities, due to high rates of contact, pivoting, acceleration and jumping activities [1]. Knee injuries may cause pain, stiffness, swelling and instability of the knee, causing impaired physical functioning and mobility. To measure concepts of activity and to acquire valuable information from patient’s perspective, Patient Reported Outcome Measure (PROM) could be used as a non-invasive measures [2].

The International Knee Documentation Committee (IKDC) Subjective Knee Form and the Knee injury and Osteoarthritis Outcome Score (KOOS) are two standardized questionnaires for patients with knee disorders. Both questionnaires measure pain, symptoms and functioning in daily and sport activities. Several studies showed that these questionnaires are valid, reliable and responsive in adult patients with a variety of knee disorders [3–5]. However, recent studies showed that the adult versions of the IKDC and KOOS are inappropriate for use in children due to a lack of comprehensibility [6]. Subsequently, the child friendly Pedi-IKDC and KOOS-Child were developed and validated [7–9].

Until now, no Dutch versions of the Pedi-IKDC and KOOS-Child were available. This study firstly translated the English Pedi-IKDC and KOOS-Child into Dutch. Secondly, the psychometric properties of these questionnaires were evaluated and compared in terms of validity, reliability, responsiveness and interpretability in children with knee disorders. The aim of this study was to determine whether the Pedi-IKDC or KOOS-Child should be used in children with a variety of knee disorders.

## Methods

### Translation procedure

The forward-backward procedure was performed according to the guidelines from the study of Beaton et al. [10]. First, two native Dutch speakers independently translated the Pedi-IKDC and the KOOS-Child from English into Dutch. Second, two native English speakers independently and blinded for the original versions translated the preliminary Dutch versions back into English. These two translations were compared with the original English versions. All inconsistencies were discussed and adjustments were made. Finally, the second preliminary Dutch versions were evaluated by the Department of Patient Communication of the Maxima Medical Centre (Eindhoven-Veldhoven, The Netherlands). After adjustments, the pre-final Dutch versions were made. Subsequently, the questionnaires were pilot tested in a heterogeneous group of 10 children, aged 10–15 years, who received treatment for orthopaedic-related knee problems. Respondents were asked about item difficulty. In case of indistinctness or incomprehensibility adjustments were made. Final Dutch versions of the Pedi-IKDC and KOOS-Child were obtained. Patients of the pilot study were not included in the validation study.

### Participants

Patients were consecutively recruited from the outpatient clinic of orthopaedics of the Maxima Medical Centre (Eindhoven-Veldhoven, The Netherlands) from February 2015 and May 2016. Patients were considered eligible if they were aged 10–18 years, had a knee injury and physical limitations. The study had the intention to include a heterogeneous patient population that was representative for children with a broad range of knee injuries. Patients were excluded if they had any comorbidity overriding their knee symptoms or had an inadequate command of the Dutch language. If necessary, parents or caregivers were allowed to assist their child in completing the questionnaires.

### Study procedure

Eligible patients visiting the outpatient clinic were asked to complete the Dutch Numeric Rating Scale (NRS) Pain, Lysholm Knee Scoring Scale, EuroQol-5 Dimension (EQ-5D), Pedi-IKDC and KOOS-Child via Research Manager (baseline (T0)). Two weeks after baseline (T1) patients answered by e-mail the anchor question ‘did your knee complaints change over the course of the past two weeks after completing the last questionnaires?’ The time point of two weeks was chosen to prevent patients from remembering the previous answer and to avoid change in knee condition. The anchor question was used as an equivalent of possible change in knee condition of the patient during the past two weeks [11]. Consequently, only patients with stable knee conditions were asked to complete the Pedi-IKDC and KOOS-Child. One year after baseline (T2) all patients were asked by e-mail to complete an anchor question, Pedi-IKDC and KOOS-Child. This time point was chosen because clinical change of the patients could be expected. If needed, patients were reminded by e-mail and telephone.

### Questionnaires

#### Pedi-IKDC

The Pedi-IKDC measures pain, symptoms and physical functioning during daily and sport activities in children and adolescents with knee-related problems [7]. A higher score represents less pain, less symptoms and a higher level of physical functioning. The Pedi-IKDC has been shown a valid, reliable and responsive questionnaire in children (aged 9–18 years) with a broad range of knee disorders [7, 9].

#### KOOS-child

The KOOS-Child evaluates short- and long-term symptoms, physical functioning during daily and sport activities and quality of life (QOL) in patients with a broad range of knee disorders. The KOOS-Child (LK 2.0) has 39 items, divided over the five subscales Symptoms, Pain, activities of daily living (ADL), Sport/play and QOL. A higher score indicates less knee-related problems. An aggregate score is not calculated since it is recommended to analyze and interpret the five dimensions separately. The study by Ortqvist et al. [12] showed good validity and reliability of the KOOS-Child in children with knee disorders.

#### NRS pain

The NRS Pain measures the level of pain at rest and during movement. The NRS Pain is a valid and reliable questionnaire in adult patients with knee osteoarthritis and a variety of musculoskeletal injuries. The NRS Pain showed strong correlations with the Faces Pain Scale Revised and Visual Analogue Scale in children, with bodily pain or postoperative pain. However, the psychometric properties of the NRS Pain in children with knee disorders have not yet been evaluated [13].

### Lysholm knee scoring scale

The Lysholm Knee Scoring Scale measures symptoms of instability and was originally developed for patients who underwent or were about to undergo a knee ligament surgery. The Lysholm Knee scale is proven to be a valid and reliable questionnaire in adolescents (12–17 years) and adults with traumatic and non-traumatic knee problems [14].

#### EQ_5D

The EQ_5D measures health-related QOL by evaluating mobility problems, self-care problems, problems during usual activities and the level of pain and anxiety. The EQ_5D has shown highly valid and reliable in adults with knee osteoarthritis or patients undergoing arthroscopic knee surgery. In children the EQ_5D has shown valid for orthopedic problems, however not specifically for knee disorders [15] .

### Psychometric properties

The psychometric properties were evaluated according to the COnsensus-based Standards for the selection of health Measurement Instruments (COSMIN) criteria [16–18].

Reliability is the degree to which a research instrument produces consistent results for patients who have not clinically changed. Based on the anchor question, patients with unstable knee condition between T0 and T1 –two weeks– were excluded from the analysis. Unstable knee conditions were defined when answered much better, better, worse or much worse. To assess the consistency of results, the test-retest reliability was measured using the two-way random effects model of Intraclass Correlation Coefficient (ICC) in absolute agreement. ICC coefficients of 0.70 or higher were considered excellent. The Standard Error of the Mean (SEM) (SEM = standard deviation (SD) difference/√number measurements) and the Smallest Detectable Change (SDC) (SDC = SEM × 1.96 × √2) were used as parameters of measurement error within the test-retest reliability.

Content validity is the degree to which the instrument is an adequate reflection of what it aims to measure. It was measured by evaluating the relevance of each item by orthopaedic surgeons/residents and patients. An item was defined as relevant if at least 75% of a subgroup found it to be relevant. The content validity of the Pedi-IKDC and KOOS-Child subscales was given a positive rating if 75% of the items were found to be relevant by both subgroups.

Construct validity is the degree to which scores of the instrument are consistent with hypothesis about the relationship to scores of other instruments. In this study, construct validity was measured by testing predefined hypotheses about the relationship of the KOOS-Child and Pedi-IKDC to other questionnaires (outlined in Table 6). A panel consisting of experts (specialist in clinimetrics and methodologist) in the field of outcome measurements in knee complaints formulated these hypotheses. These hypotheses were tested by calculating Pearson’s correlation coefficient between scores at T0 of the Pedi-IKDC, KOOS-Child subscales, NRS Pain, Lysholm scale and EQ-5D. Questionnaires measuring similar constructs were expected to correlate 0.5 or higher. In contrast, questionnaires focussing on different constructs were expected to correlate 0.35 or less. Direction of relation was taken into account by means of sign of expected correlation. The construct validity of the Pedi-IKDC and KOOS-Child subscales was considered good if at least 75% of the predefined hypotheses were accepted.

Interpretability is the degree to which one can assign qualitative meaning to a score. The interpretability was assed at T0 by examining the distribution of the Pedi-IKDC score and KOOS-Child subscale scores, including the mean, SD, minimum, maximum, median and floor and ceiling effects. Floor and ceiling effects were considered present if ≥15% of the patients scored the Pedi-IKDC or a KOOS-Child subscale with respectively 0–5 or 95–100. The interpretability of the Pedi-IKDC and KOOS-Child subscales was given a positive rating if floor or ceiling effects were absent.

Responsiveness is the ability of an instrument to detect change over time. The responsiveness was evaluated by comparing the change scores of the Pedi-IKDC and KOOS-Child subscales between baseline (T0) and 1 year (T2). Effect size (ES) was calculated as follows: (mean T2 – mean baseline scale)/SD of baseline scale. Standardized response mean (SRM) was calculated as follows: (mean T2 – mean baseline scale)/SD of change in scale. Hypotheses about the expected effect size and standardized response mean of the KOOS-Child and the Pedi-IKDC subjective were formulated (outlined in Table 6). Hypotheses were formulated separately for patients reporting no change and for patients who reported a difference in their experience knee condition (improvement or worsening). In creating the hypotheses, ES of ≥0.2 were interpreted as small, ≥0.4 as medium, ≥0.8 as large. Responsiveness was scored as adequate if minimally 75% of the hypotheses were confirmed.

A sample size of at least 50 patients is recommended for good test-retest reliability, construct validity, interpretability and responsiveness and > 10 respondents for excellent content validity [19]. All questionnaires were collected using the software program Research Manager Version 4.7.2.4. Data were analysed with SPSS version 22 (IBM SPSS Statistics). A significance level of *p* ≤ 0.05 was applied for all statistical analysis.

## Results

Of the included 101 patients with knee symptoms, 100 patients completed the T0 questionnaires. 1 patient did not complete the baseline questionnaire. Table 1 represent the baseline characteristics of included patients. Due to a loss to follow up of 42 patients, 59 patients were included for the test-retest measurement at T1 for the Pedi-IKDC and 53 patients for the KOOS-Child subscales. Patients were excluded for the test-retest measurement due to missing responses (*n* = 22) or an unstable knee condition (*n* = 20). The mean interval between the T0 and T1 measurement from the patients with a stable knee condition was 19 days (SD = 6.95). At T2, 71 patients were included for measuring responsiveness. Due to missing responses 30 patients were lost to follow up.

**Table 1:** Patient characteristics at T0

Characteristic	Value
Male sex, n (%)	34 (33.7)
Mean age, years (SD)	15.0 (1.85)
Mean BMI, kg/m^2^ (SD)	20.70 (3.37)
Injury left side, n (%)	52 (51.5)
First visit, n (%)	51 (49.5)
First injury, n (%)	78 (77.2)
Injury duration, n (%)a	
0–1 months	15 (14.9)
1–3 months	21 (20.8)
4–6 months	22 (21.8)
> 6 months	43 (42.6)
Diagnosis, n (%)	
Anterior cruciate ligament rupture	24 (23.8)
• Solitary	14 (13.9)
• combined with anterolateral injury	3 (3)
• combined with medial collateral ligament injury	2 (2)
• combined with medial meniscus tear	5 (5)
Meniscal tear	7 (6.9)
Collateral ligament injury	2 (2)
Patellofemoral pain syndrome	22 (21.8)
Contusion/distorsion	11 (10.9)
Osgood-Schlatter	4 (4)
Chondropathy	10 (9.9)
Other	21 (20.8)
• Baker’s cyst	1 (1)
• Bone bruise	1 (1)
• Lyme arthritis	1 (1)
• Bone bruise	1 (1)
• Patellar tendinopathy	3 (3)
• Laxity anterior cruciate ligament	1 (1)
• Osteochondritis dissecans	5 (5)
• Patella luxation	1 (1)
• Plica syndrome	2 (2)
• Reumatoid arthritis	1 (1)
• Sinding Larson	1 (1)
• Unknown origin	3 (3)

a percentages should not be summed up as number are rounded to one decimal place n, number of patients; *SD* Standard Deviation; cm, centimeter, *kg* kilogram, *m* meter, *BMI* Body Mass Index

### Translation process

Overall, the forward-backward translation revealed minor differences in translation. Small adaptations in structure and tense were made. No cross-cultural adaptations were necessary.

### Test-retest reliability (Table 2)

The test-retest reliability of the Pedi-IKDC and KOOS-Child subscales were given a positive rating, as all ICC values were higher than 0.7 (Pedi-IKDC ICC: 0.9, KOOS-Child ICC 0.8–0.9). The SEM of the Pedi-IKDC was 8.6 and of the KOOS-Child subscales ranged from 8.9–16.9. The SDC of the Pedi-IKDC was 23.8 and of the KOOS-Child subscales ranged from 24.7–46.9.

**Table 2:** Test-retest reliability of the Pedi-IKDC and KOOS-Child subscales (*n* = 59)

	Mean score T0 (SD)	Mean score T1 (SD)	Mean difference (SD)	ICC	95% CI	SEM	SDC
Pedi-IKDC	54.5 (19.7)	55.4 (20.0)	0.9 (12.1)	0.9	0.8 to 0.9	8.6	23.8
KOOS-Child Symptoms	68.2 (22.8)	70.3 (19.8)	2.1 (14.5)	0.9	0.8 to 0.9	10.2	28.4
KOOS-Child Pain	62.0 (23.8)	63.1 (23.1)	1.1 (13.9)	0.9	0.8 to 1.0	9.9	27.3
KOOS-Child ADL	77.8 (21.1)	78.0 (22.5)	0.2 (12.6)	0.9	0.8 to 1.0	8.9	24.7
KOOS-Child Sport/play	41.6 (30.3)	46.6 (30.9)	5.0 (23.9)	0.8	0.7 to 0.9	16.9	46.9
KOOS-Child QOL	47.9 (22.1)	47.3 (24.0)	0.6 (14.9)	0.9	0.8 to 0.9	10.5	29.2

*n* number of patients, *SD* Standard Deviation, *ICC* Intraclass Correlation Coefficient, *CI* Confidence Interval, *SEM* Standard Error of the Mean, *SDC* Smallest Detectable Change, *IKDC* The International Knee Documentation Committee Subjective Knee Form, *KOOS* Knee injury and Osteoarthritis Outcome Score, *ADL* Activities of Daily Living, *QOL* Quality Of Life

### Content validity (Table 3)

The Pedi-IKDC and all KOOS-Child subscales were all found relevant by patients (all > 75%). The orthopaedic surgeons rated the Pedi-IKDC and KOOS-Child subscales as relevant (all > 75%), except the KOOS-Child ADL (64%). Furthermore, we received feedback from both groups that the KOOS-Child has a higher patient burden, since it has multiple subscales.

**Table 3:** Content validity of the Pedi-IKDC and KOOS-Child subscales

	Relevant items / total items (%)	
	Orthopaedic surgeons (*n* = 18)	Patients (*n* = 18)
Pedi-IKDC	**20/21 (95%)**	**20/21 (95%)**
KOOS-Child Symptoms	**6/7 (86%)**	**7/7 (100%)**
KOOS-Child Pain	**7/8 (88%)**	**7/8 (88%)**
KOOS-Child ADL	7/11 (64%)	**9/11 (82%)**
KOOS-Child Sport/play	**7/7 (100%)**	**7/7 (100%)**
KOOS-Child QOL	**5/6 (83%)**	**6/6 (100%)**

Relevant questionnaire/subscales (< 75%) are represented in bold IKDC, The International Knee Documentation Committee Subjective Knee Form; *KOOS* Knee injury and Osteoarthritis Outcome Score, *ADL* Activities of Daily Living, *QOL* Quality Of Life, *n* number of patients

### Construct validity (Table 4)

The Pedi-IKDC showed good construct validity, as 75% of the predefined hypotheses were accepted. The KOOS-Child subscales Symptoms, Pain, ADL and Sport/play showed good construct validity, as 75% of the predefined hypotheses were accepted.

**Table 4:** Construct validity of the Pedi-IKDC and KOOS-Child subscales (*n* = 100)

	Pedi-IKDC	KOOS Symptoms	KOOS Pain	KOOS ADL	KOOS Sport/play	KOOS QOL
Pedi-IKDC	–					
Pearson *r*		0.69	0.84	0.83	0.80	0.76
(Predefined *r*)		(≥ 0.5)	(≥ 0.5)	(≥ 0.5)	(≥ 0.5)	(≤ 0.35)
Hypothesis confirmed		Yes	Yes	Yes	Yes	No
Lysholm						
Pearson *r*	0.71	0.70	0.71	0.66	0.56	0.57
(Predefined *r*)	(≥ 0.5)	(≥ 0.5)	(≥ 0.5)	(≥ 0.5)	(≥ 0.5)	(≤ 0.35)
Hypothesis confirmed	Yes	Yes	Yes	Yes	Yes	No
NRS Pain						
Pearson *r*	−0.70	−0.44	−0.71	−0.62	−0.54	−0.52
(Predefined *r*)	(≤ −0.5)	(≤ −0.5)	(≤ − 0.5)	(≤ − 0.5)	(≤ − 0.5)	(≥ − 0.35)
Hypothesis confirmed	Yes	Yes	Yes	Yes	Yes	No
EQ_5D						
Pearson *r*	0.53	0.42	0.52	0.50	0.45	0.53
(Predefined *r*)	(≤ 0.35)	(≤ 0.35)	(≤ 0.35)	(≤ 0.35)	(≤ 0.35)	(≥ 0.5)
Hypothesis confirmed	No	No	No	No	No	Yes
Confirmed hypothesis *n* (%)	6/8 (75%)	3/4 (75%)	3/4 (75%)	3/4 (75%)	3/4 (75%)	1/4 (25%)

All Pearson’s correlation coefficients were significant for the Pedi-IKDC and KOOS-Child subscales (*p* < 0.01)

IKDC, The International Knee Documentation Committee Subjective Knee Form; KOOS Knee injury and Osteoarthritis Outcome Score; ADL, Activities of Daily Living; QOL, Quality Of Life; NRS, Numeric Rating Scale pain; Lysholm, Lysholm Knee Scoring Scale; EQ-5D, EuroQol-5 Dimension; Pearson *r,* correlation coefficient; n, number of hypothesis

### Interpretability (Table 5)

The Pedi-IKDC and KOOS-Child Symptoms, Pain and QOL showed acceptable floor and ceiling effects (score between 5 and 95). Ceiling effects were found in the KOOS-Child ADL, as 24.8% of the patients had the maximal score of > 95. Floor effects were found in the KOOS-Child Sport/play subscale, as 17.8% of the patients had the minimal score of < 5.

**Table 5:** Interpretability of the Pedi-IKDC and KOOS-Child subscales at T0 (*n* = 100)

	Mean score T0 (SD)	Minimum	Maximum	Median	Floor effects worst score (%)	Ceiling effects best score (%)
Pedi-IKDC (*n* = 54)	53.2 (19.3)	12.0	95.7	50.0	0	1.0
KOOS-Child Symptoms (*n* = 53)	67.7 (19.7)	14.3	100.0	71.4	0	5.0
KOOS-Child Pain (*n* = 53)	62.6 (22.6)	6.3	100.0	65.6	0	5.0
KOOS-Child ADL (*n* = 53)	77.9 (20.6)	15.9	100.0	81.8	0	25.0
KOOS-Child Sport/play (*n* = 53)	38.0 (27.6)	0.0	100.0	39.3	18.0	2.0
KOOS-Child QOL (*n* = 53)	47.2 (21.6)	0.0	95.8	43.8	1.0	1.0

*n* number of patients, *SD* Standard Deviation, *IKDC* The International Knee Documentation Committee Subjective Knee Form, *KOOS* Knee injury and Osteoarthritis Outcome Score, *ADL* Activities of Daily Living, *QOL* Quality Of Life

### Responsiveness (Table 6)

The Pedi-IKDC showed good responsiveness, as 75% of the predefined hypotheses were accepted. The KOOS-Child subscales ADL and QOL showed good responsiveness, as all hypotheses were accepted. The KOOS-Child subscales Symptoms, Pain, ADL and Sport/play showed moderate responsiveness, as 50% of the hypotheses were accepted.

**Table 6:** Responsiveness of the Pedi-IKDC and KOOS-Child subscales *N* = 71

		Pedi-IKDC	KOOS Symptoms	KOOS Pain	KOOS ADL	KOOS Sport/play	KOOS QOL
ES	*No change (n = 22)*	0.24	0.29	0.27	0.13	0.28	− 0.07
	(Hypothesis)	(− 0.2 to 0.2)	(− 0.2 to 0.2)	(− 0.2 to 0.2)	(− 0.2 to 0.2)	(− 0.2 to 0.2)	(− 0.2 to 0.2)
	Confirmed?	No	No	No	Yes	No	Yes
	*Improvement (n = 42)*	1.5	0.84	1.26	0.83	1.57	1.24
	(Hypothesis)	(≥0.4)	(≥0.4)	(≥0.4)	(≥0.4)	(≥0.4)	(≥0.4)
	AND						
	*Worsening (n = 7)*	− 0.66	− 0.44	−0.43	−0.75	− 1.23	−0.83
	(Hypothesis)	(≤ − 0.4)	(≤ − 0.4)	(≤ − 0.4)	(≤ − 0.4)	(≤ − 0.4)	(≤ − 0.4)
	Confirmed?	Yes	Yes	Yes	Yes	Yes	Yes
SRM	*No change (n = 22)*	0.20	0.29	0.27	0.11	0.23	−0.06
	(Hypothesis)	(−0.2 to 0.2)	(− 0.2 to 0.2)	(− 0.2 to 0.2)	(− 0.2 to 0.2)	(− 0.2 to 0.2)	(− 0.2 to 0.2)
	Confirmed?	Yes	No	No	Yes	No	Yes
	*Improvement (n = 42)*	1.25	0.69	1.06	0.74	1.13	0.82
	(Hypothesis)	(≥0.4)	(≥0.4)	(≥0.4)	(≥0.4)	(≥0.4)	(≥0.4)
	AND						
	*Worsening (n = 7)*	−0.41	− 0.76	− 1.03	−0.85	−0.66	− 1.82
	(Hypothesis)	(≤ − 0.4)	(≤ − 0.4)	(≤ − 0.4)	(≤ − 0.4)	(≤ − 0.4)	(≤ − 0.4)
	Confirmed?	Yes	Yes	Yes	Yes	Yes	Yes
	Confirmed hypothesis n (%)	3/4 (75%)	2/4 (50%)	2/4 (50%)	4/4 (100%)	2/4 (50%)	4/4 (100%)

*ES* Effect Size, *SRM* Standardized Response Mean, *IKDC* The International Knee Documentation Committee Subjective Knee Form, *KOOS* Knee injury and Osteoarthritis Outcome Score, *ADL* Activities of Daily Living, *QOL* Quality Of Life, *NRS* Numeric Rating Scale pain, Lysholm, Lysholm Knee Scoring Scale, *EQ-5D* EuroQol-5 Dimension, *n* number

## Discussion

In the present study the Pedi-IKDC and KOOS-Child were translated into Dutch and the psychometric properties of these questionnaires were evaluated in children with knee symptoms.

This study found good test-retest reliability for the Pedi-IKDC (ICC 0.9) and KOOS-Child subscales (ICC 0.8–0.9). In addition, our study showed no significant differences of the Pedi-IKDC and KOOS-Child subscale mean scores during the test-retest period. This implies that the questionnaires are reproducible in children with stable knee complaints. ICC values found in this study are similar to those of the original Pedi-IKDC and KOOS-Child subscales [7, 12]. The SEM and SDC values in this study were higher than those presented in other studies. [9] [12] This can partly be due to the smaller sample size used in our study, as the sample size directly influences the magnitude of the SEM and SDC [20]. Higher SEM and SDC scores indicate that large differences in scores are needed to conclude that a true change occurred. This is a well-known negative aspect of questionnaires [20].

Our results showed a good content validity of the Pedi-IKDC and all KOOS-Child subscales. Content validity of these questionnaires had not been measured in children before. Previous studies showed good content validity of the IKDC and KOOS subscales symptoms, Sport/play and QOL in adults [21, 22]. Hambly et al. [23] and van Meer et al. [22] showed low content validity for KOOS Pain and ADL.

Construct instead of criterion validity was assessed in this study, since no gold standard was available. Construct validity for the Pedi-IKDC was found to be good. Kocher et al. [7], showed similar results. For the KOOS-Child Symptoms, Pain, ADL, Sport/play the construct validity was found good. This is in accordance with Ortqvist et al., except for the KOOS-Child QOL, which was found good in their study [12]. This can be due to the fact that they used different questionnaires in comparison [12].

No floor or ceiling effects were found in the Pedi-IKDC. This is in accordance with the study of Kocher et al. [7]. There were ceiling effects in the KOOS-Child ADL and floor effects in the Sport/play. The study of Ortqvist et al. [12] also reported a ceiling effect in the KOOS-Child ADL subscale. Ceiling and floor effects might have occurred earlier in this study due to our cut off point of 5 and 95, instead of 0 and 100. This cut off point was chosen to ensure no missing items when given an answer in the lower or upper end of the scale.

The Pedi-IKDC showed good responsiveness, previous studies found similar results [7, 9]. The KOOS-Child showed good responsiveness in the subscale ADL and QOL and moderate responsiveness in subscale Symptoms, Pain and Sport/play. Ortqvist et al. [12] found similar results.

This study has several limitations. Firstly, the NRS Pain, Lysholm scale and EQ-5D have been validated, however there is limited evidence when used in children. Secondly, the definition of the hypothesis is an arbitrary procedure and the percentage of rejected hypothesis depends on the number of a priori hypotheses. Thirdly, all hypotheses counted equally in the confirmation of the construct validity. While certain hypothesis should have had more weight than others when they had stronger correlations. Furthermore, risk of bias could have been occurred since parents could assist their children by explaining questions when completing the questionnaires. In our study at T1, 34% of the participants reported that their parents assisted. Dietvorst et al. reported that children’s self-reports of functioning are not equal to reports by proxy respondents [24]. Therefore, the allowed assisting might have been a potential source of bias.

The strength of this study is the inclusion of a large sample size and a heterogeneous population. This makes the results generalizable to a large group of children with a variety of knee disorders. In addition, psychometric properties are calculated and interpreted using the renowned COSMIN guidelines, which makes this study highly reproducible for validation of questionnaires in other populations/languages. The availability of validated questionnaires in different languages becomes more and more of importance because of large-scale initiatives including multiple countries together addressing a clinical question. An example is the recently, by the European Society for Sports Traumatology, Knee Surgery and Arthroscopy (ESSKA), launched Paediatric Anterior Cruciate Ligament Initiative (PAMI). Furthermore, our results are in accordance with the review of Dietvorst et al. This review showed that the Pedi-IKDC is a valid, reliable and responsive questionnaire. The KOOS-Child was considered as an alternative PROM but was not as well studied [24].

## Conclusion

The Pedi-IKDC and KOOS-Child were successfully translated into Dutch. Based on psychometric properties, the Pedi-IKDC showed better results than the KOOS-Child Furthermore, the Pedi-IKDC is shorter and therefore easier to apply in daily practice. As such, the Pedi-IKDC is recommended to be used in children with knee disorders for clinical and research purposes.

## Abbreviations

ADL: Activities of daily living
COSMIN: COnsensus-based Standards for the selection of health Measurement INstruments
EQ-5D: EuroQol-5 Dimension
ES: Effect size
ICC: Intraclass correlation coefficient
IKDC: International Knee Documentation Committee
KOOS: Knee Injury and Osteoarthritis Outcome Score
METC: Medical Ethics Committee
NRS: Numeric Rating Scale
PROM: Patient Reported Outcome Measure
QOL: Quality of life
SD: Standard deviation
SDC: Smallest detectable change
SEM: Standard error of measurement
SRM: Standardizes response mean

## References

[1] Kerssemakers SP, Fotiadou AN, de Jonge MC, Karantanas AH, Maas M (2009). Sport injuries in the paediatric and adolescent patient: a growing problem. Pediatr Radiol.

[2] Bremander AB, Dahl LL, Roos EM (2007). Validity and reliability of functional performance tests in meniscectomized patients with or without knee osteoarthritis. Scand J Med Sci Sports.

[3] Collins NJ, Misra D, Felson DT, Crossley KM, Roos EM (2011). Measures of knee function: international knee documentation committee (IKDC) subjective knee evaluation form, knee injury and osteoarthritis outcome score (KOOS), knee injury and osteoarthritis outcome score physical function short form (KOOS-PS), knee outcome survey activities of daily living scale (KOS-ADL), Lysholm knee scoring scale, Oxford knee score (OKS), Western Ontario and McMaster universities osteoarthritis index (WOMAC), activity rating scale (ARS), and Tegner activity score (TAS). Arthritis Care Res (Hoboken).

[4] Irrgang JJ, Anderson AF, Boland AL, Harner CD, Kurosaka M, Neyret P (2001). Development and validation of the international knee documentation committee subjective knee form. Am J Sports Med.

[5] Roos EM, Roos HP, Lohmander LS, Ekdahl C, Beynnon BD (1998). Knee injury and osteoarthritis outcome score (KOOS)--development of a self-administered outcome measure. J Orthop Sports Phys Ther.

[6] Iversen MD, von Heideken J, Farmer E, Rihm J, Heyworth BE, Kocher MS (2016). Validity and comprehensibility of physical activity scales for children with sport injuries. J Pediatr Orthop.

[7] Kocher MS, Smith JT, Iversen MD, Brustowicz K, Ogunwole O, Andersen J (2011). Reliability, validity, and responsiveness of a modified international knee documentation committee subjective knee form (Pedi-IKDC) in children with knee disorders. Am J Sports Med.

[8] Ortqvist M, Roos EM, Brostrom EW, Janarv PM, Iversen MD (2012). Development of the knee injury and osteoarthritis outcome score for children (KOOS-child): comprehensibility and content validity. Acta Orthop.

[9] Jacobsen J. S., Knudsen P., Fynbo C., Rolving N., Warming S. (2015). Reproducibility and responsiveness of a Danish Pedi-IKDC subjective knee form for children with knee disorders. Scandinavian Journal of Medicine & Science in Sports.

[10] Beaton DE, Bombardier C, Guillemin F, Ferraz MB (2000). Guidelines for the process of cross-cultural adaptation of self-report measures. Spine (Phila Pa 1976).

[11] Jaeschke R, Singer J, Guyatt GH (1989). Measurement of health status. Ascertaining the minimal clinically important difference. Control Clin Trials.

[12] Ortqvist M, Iversen MD, Janarv PM, Brostrom EW, Roos EM (2014). Psychometric properties of the knee injury and osteoarthritis outcome score for children (KOOS-child) in children with knee disorders. Br J Sports Med.

[13] Hawker GA, Mian S, Kendzerska T, French M (2011). Measures of adult pain: visual analog scale for pain (vas pain), numeric rating scale for pain (nrs pain), mcgill pain questionnaire (mpq), short-form mcgill pain questionnaire (sf-mpq), chronic pain grade scale (cpgs), short form-36 bodily pain scale (sf-36 bps), and measure of intermittent and constant osteoarthritis pain (icoap). Arthritis Care Res.

[14] Lysholm J, Gillquist J (1982). Evaluation of knee ligament surgery results with special emphasis on use of a scoring scale. Am J Sports Med.

[15] Vitale MG, Levy DE, Johnson MG, Gelijns AC, Moskowitz AJ, Roye BP (2001). Assessment of quality of life in adolescent patients with orthopaedic problems: are adult measures appropriate?. J Pediatr Orthop.

[16] Mokkink LB, Terwee CB, Patrick DL, Alonso J, Stratford PW, Knol DL (2010). The COSMIN checklist for assessing the methodological quality of studies on measurement properties of health status measurement instruments: an international Delphi study. Qual Life Res.

[17] Mokkink LB, Terwee CB, Patrick DL, Alonso J, Stratford PW, Knol DL (2010). The COSMIN study reached international consensus on taxonomy, terminology, and definitions of measurement properties for health-related patient-reported outcomes. J Clin Epidemiol.

[18] Terwee CB, Bot SD, de Boer MR, van der Windt DA, Knol DL, Dekker J (2007). Quality criteria were proposed for measurement properties of health status questionnaires. J Clin Epidemiol.

[19] Terwee CB, Mokkink LB, Knol DL, Ostelo RW, Bouter LM, de Vet HC (2012). Rating the methodological quality in systematic reviews of studies on measurement properties: a scoring system for the COSMIN checklist. Qual Life Res.

[20] de Vet HC, Bouter LM, Bezemer PD, Beurskens AJ (2001). Reproducibility and responsiveness of evaluative outcome measures. Theoretical considerations illustrated by an empirical example. Int J Technol Assess Health Care.

[21] Haverkamp D, Sierevelt IN, Breugem SJ, Lohuis K, Blankevoort L, van Dijk CN (2006). Translation and validation of the Dutch version of the international knee documentation committee subjective knee form. Am J Sports Med.

[22] van Meer BL, Meuffels DE, Vissers MM, Bierma-Zeinstra SM, Verhaar JA, Terwee CB (2013). Knee injury and osteoarthritis outcome score or international knee documentation committee subjective knee form: which questionnaire is most useful to monitor patients with an anterior cruciate ligament rupture in the short term?. Arthroscopy..

[23] Hambly K, Griva K (2008). IKDC or KOOS? Which measures symptoms and disabilities most important to postoperative articular cartilage repair patients?. Am J Sports Med.

[24] Dietvorst M, Reijman M, van Groningen B, van der Steen MC, Janssen RPA. PROMs in paediatric knee ligament injury: use the Pedi-IKDC and avoid using adult PROMs. Knee Surg Sports Traumatol Arthrosc. 2017. 10.1007/s00167-017-4687-3. Epub 2017 10.1007/s00167-017-4687-3 28929208

